# Problems and progress in the growth and understanding of kagome materials

**DOI:** 10.1063/4.0000924

**Published:** 2025-10-27

**Authors:** Michael A McGuire

**Affiliations:** Oak Ridge National Laboratory, Oak Ridge, TN, USA

## Abstract

The connectivity of the kagome network has long provided a source of magnetic frustration in the search for interesting quantum magnets, and more recently a source of electronic frustration for the development of topological and correlated materials. In this presentation, I will share some of our ongoing work on materials containing kagome or distorted kagome networks. This includes the double-layer kagome metal V3Sb2, which hosts a charge density wave like phase transition, as well as members of the ZrNiAl structure type, with a distorted kagome net of transition metals. The content will focus on some surprises and complications associated with the synthesis, crystal growth, and structural characterization of several materials, as well as some interesting new phase transitions and physical behaviors we have observed.

